# Analysis of Human Accelerated DNA Regions Using Archaic Hominin Genomes

**DOI:** 10.1371/journal.pone.0032877

**Published:** 2012-03-07

**Authors:** Hernán A. Burbano, Richard E. Green, Tomislav Maricic, Carles Lalueza-Fox, Marco de la Rasilla, Antonio Rosas, Janet Kelso, Katherine S. Pollard, Michael Lachmann, Svante Pääbo

**Affiliations:** 1 Max Planck Institute for Evolutionary Anthropology, Leipzig, Germany; 2 Department of Biomolecular Engineering, University of California Santa Cruz, Santa Cruz, California, United States of America; 3 Institute of Evolutionary Biology, Consejo Superior de Investigaciones Científicas, Universitat Pompeu Fabra, Barcelona, Spain; 4 Área de Prehistoria, Departamento de Historia, Universidad de Oviedo, Oviedo, Spain; 5 Departamento de Paleobiología, Museo Nacional de Ciencias Naturales, Consejo Superior de Investigaciones Científicas, Madrid, Spain; 6 Gladstone Institutes, University of California San Francisco, San Francisco, California, United States of America; 7 Division of Biostatistics and Institute of Human Genetics, University of California San Francisco, San Francisco, California, United States of America; University of Uppsala, Sweden

## Abstract

Several previous comparisons of the human genome with other primate and vertebrate genomes identified genomic regions that are highly conserved in vertebrate evolution but fast-evolving on the human lineage. These human accelerated regions (HARs) may be regions of past adaptive evolution in humans. Alternatively, they may be the result of non-adaptive processes, such as biased gene conversion. We captured and sequenced DNA from a collection of previously published HARs using DNA from an Iberian Neandertal. Combining these new data with shotgun sequence from the Neandertal and Denisova draft genomes, we determine at least one archaic hominin allele for 84% of all positions within HARs. We find that 8% of HAR substitutions are not observed in the archaic hominins and are thus recent in the sense that the derived allele had not come to fixation in the common ancestor of modern humans and archaic hominins. Further, we find that recent substitutions in HARs tend to have come to fixation faster than substitutions elsewhere in the genome and that substitutions in HARs tend to cluster in time, consistent with an episodic rather than a clock-like process underlying HAR evolution. Our catalog of sequence changes in HARs will help prioritize them for functional studies of genomic elements potentially responsible for modern human adaptations.

## Introduction

To detect functionally relevant genomic features that underwent positive selection in humans since the separation from their common ancestor with chimpanzees and bonobos, several authors identified genomic regions that are conserved among vertebrates but have accumulated substitutions on the human lineage at an accelerated rate [1], [2], [3], [4]. Here, we refer collectively to these regions as “human accelerated regions” (HARs).

The sequence conservation in HARs suggests that they are subject to functional constraints, while the increased rate of substitutions on the human lineage suggests that their function may have changed in humans. To date, two HARs have been studied in detail with respect to function. One of these (HAR1 in [3]) is part of an RNA gene that is expressed in Cajal-Retzius neurons in the developing human cortex between gestational week 7 and 19, while another (HAR2 in [4]) acts as an enhancer of gene expression in transgenic mice and has limb expression in humans which is not seen in chimpanzees and rhesus macaques [5]. However, it is unclear to what extent all HARs are functionally important. Although some HARs are located near genes that encode transcription factors and other DNA-binding proteins [6], genes involved in neuronal cell adhesion [4], and genes containing polymorphisms correlated with changes in gene expression [1], it has been noted that they are not enriched for cis-regulatory elements [1], [2], [3], [4] although other conserved non-coding sequences are so [7].

It has been noted that nucleotide substitutions in HARs show an excess of A/T to G/C substitutions [6], [8]. This is reminiscent of GC-biased gene conversion (BGC), a non-adaptive process associated with recombination in eukaryotes. BGC is the non-reciprocal copying of a stretch of DNA from one chromosome into the other [9] and favors fixation of GC alleles over AT alleles in yeast [10] and presumably in primates [11]. Since recombination, and therefore BGC, tends to be localized to recombination hotspots [12], and since these often shift their locations over short evolutionary times in primates [13], [14], an increase in the rate of substitutions in a genomic region in a certain evolutionary lineage may be due to a recombination hotspot that has appeared in that region and lineage. Thus, it has been hypothesized that repeated events of BGC are the source of human-specific substitutions in many HARs [8], [9].

One limitation in investigating HARs and their potential role in recent human evolution is that it is unknown when during the 5–7 million years since the divergence of the human lineage from the chimpanzee lineage substitutions in HARs occurred. The genomes of extinct close relatives of present-day humans offer the possibility to determine when such substitutions took place. Recently, two draft genome sequences of archaic hominins have been determined: Neandertals [15] and Denisovans [16]. Neandertals and Denisovans were sister groups whose DNA sequences diverged from those of modern humans on average about 800,000 years ago. These two draft genomes, of about 1.3- and 1.9-fold genomic coverage, respectively, provide a means to determine if nucleotide substitutions observed in human DNA sequences occurred before or after the divergence of the common ancestor of Neandertals and Denisovans from the lineage leading to modern humans. However, of all nucleotide substitutions assigned to the human lineage since the divergence of humans from chimpanzees, a total of only 30 and 40% are covered by at least one sequenced DNA fragment in the Neandertal and Denisovan genomes, respectively [15], [16]. This is below the theoretical expectation from random fragmentation and sampling of DNA (63 and 85%, respectively) [17] and is likely to be due to the fact that preservation, recovery, and mapping of ancient DNA fragments are not random.

To provide more complete coverage of particular regions of interest, several approaches that are able to recover specific genomic regions have been developed or applied to ancient DNA [18], [19], [20]. Here, we use hybridization capture on microarrays [20], [21] to capture and sequence HARs from a different Neandertal to that from which the genome was sequenced: a ∼49,000-year-old male Neandertal (Inv. Number Sidrón 1253) from El Sidrón Cave, Asturias, Spain [22], [23]. We combine this sequence data with sequence information from the Neandertal and Denisovan draft genomes in order to study the evolution of HARs.

## Results

### Capture of El Sidrón Neandertal DNA

**Figure 1:**
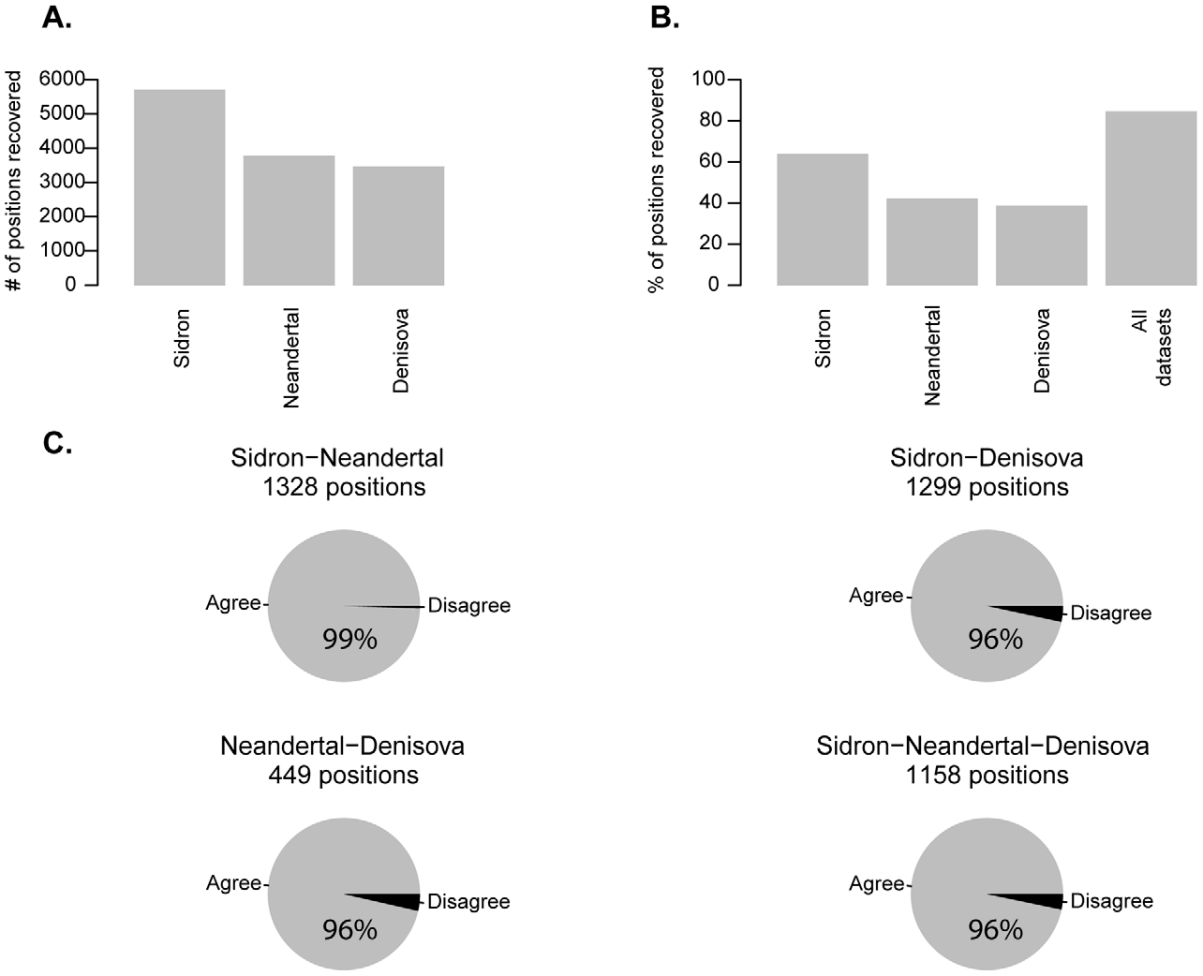
Substitutions in HARs recovered from the array capture experiment (Sidrón) and two published hominin genomes (Neandertal and Denisova). (**A**) Number of substitutions on the human lineage recovered in each dataset. (**B**) Fraction of substitutions recovered in each dataset and all datasets together. (**C**) Pie charts of substitutions from all possible overlaps among datasets (agree: datasets have same state; disagree: at least two datasets differ).

We designed an Agilent oligonucleotide capture array covering 2,613 HARs (total of ∼1 Mb) identified in four studies [1], [2], [3], [4] and used this array to capture Neandertal DNA sequences from 10 DNA libraries from a Neandertal from El Sidrón Cave, Asturias, Spain (Sidrón 1253) [22], [23]. The captured DNA fragments were sequenced on the Illumina GAIIx platform and mapped [24] to the human reference genome. From the capture of these Sidrón libraries we obtained at least one sequencing read covering 5,711 (63%) of the human lineage-specific substitutions within HARs. From the Neandertal [15] and Denisovan [16] draft genomes, we retrieved 3,779 (42%) and 3,468 (38%) such positions, respectively. Combining the three data sets produced a total coverage of 7,566 (84%) positions with human lineage-specific changes within HARs (Fig. 1a–b).

To determine the archaic state at each position, we filtered the Sidrón Neandertal data for PCR duplicates and used a maximum likelihood approach to generate consensus sequences from overlapping, independent fragments and join them in “minicontigs”, as described previously [15], [16]. In order to avoid biases induced by sequencing errors, we considered only positions where the Neandertal carried the derived (human-like) or the ancestral (chimpanzee-like) nucleotide. We refer to positions where at least one archaic hominin carries the nucleotide seen in the human reference genome as “old alleles” since they are likely to have been present in the common ancestors of present-day humans and Neandertals and Denisovans. We refer to positions where neither the chimpanzees nor any available archaic hominin genome carries the human nucleotide as “recent alleles” in the sense that they had not come to fixation in the common ancestor of the archaic hominins and modern humans. Therefore, they represent substitutions that occurred recently along the human evolutionary lineage. In all further analyses we use positions recovered from one or more of the three data sets: the Sidrón Neandertal, the Neandertal genome and the Denisova genome. For positions that overlapped among datasets the agreement in terms of whether old or recent alleles were seen was 96–99% (Fig. 1c). We therefore restricted the analyses to the positions where all datasets agreed.

### Estimates of human DNA contamination

One of the major challenges when working with ancient hominin DNA is the contamination of extracts with contemporary human DNA. Although this specimen has been excavated using procedures that minimize contamination of human DNA [25] and processed using laboratory procedures designed limit and detect contamination [26], it is nevertheless essential to estimate the level of human DNA contamination directly from the sequencing data. Here, we used two approaches to estimate modern human contamination in the Sidrón dataset based on mitochondrial DNA (mtDNA) and autosomal positions

The first approach used mtDNA. The mtDNA sequence from the Sidrón 1253 bone has been previously determined [18] and differed from almost all (99%) present-day human mtDNAs at 130 positions. We estimated the level of modern human mtDNA contamination by classifying each Sidrón mtDNA fragment carrying at least one of the 130 positions as modern human-like or Neandertal-like [27]. From a total of 127,610 informative fragments, 473 were modern human-like, indicating an mtDNA contamination level of 0.36%, with a 95% binomial confidence interval between 0.33–0.4%.

The second test estimated modern human contamination using autosomal positions, where present-day humans are fixed for the derived allele as judged from the present-day human polymorphism database dbSNP (v. 131). Briefly, for every such position, the Neandertal individual is expected to be homozygous (for the ancestral or the derived allele) or heterozygous, yielding an expectation of seeing only one allele (if the Sidrón individual was homozygous) or a draw of the two alleles with equal probabilities (if he was heterozygous). Contamination or sequencing errors will skew these expectations. We applied a maximum likelihood model that exploits this aspect of the data [20] and estimated an upper bound of contamination of <1% (Fig. S1). Thus, both a mtDNA, which is not model dependent, and an autosomal-based estimates, which is directly relevant for the data analyzed, suggest that the contamination is low.

### HAR substitutions along the human lineage

**Figure 2:**
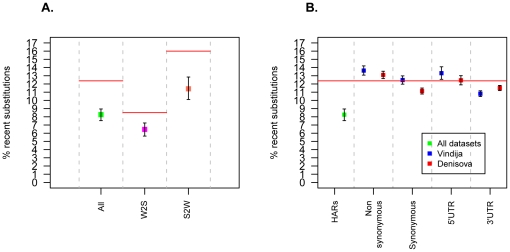
Percentage of substitutions in HARs and other functional categories classified as recent. (**A**) Recent substitutions in HARs. Boxplots represent all substitutions and substitutions from A/T to G/C (W2S) and from G/C to A/T (S2W) base pairs, respectively. Recent substitutions are defined as those found in modern humans but none of the ancient hominins (determined using Sidrón, Neandertal and Denisova datasets). The red lines show the genome-wide averages. (**B**) Recent substitutions in different functional categories. Boxplots represent HARs and other categories of genomic elements. Substitutions in HARs are defined as in (a) while substitutions in other categories are defined using either the Neandertal or Denisova genome. In both panels, the red line shows the genome-wide average percentage of recent substitutions (12%). Error bars for HARs are 95% confidence intervals calculated from an empirical bootstrap distribution (1,000 iterations). Error bars for other functional categories are 95% binomial confidence intervals. Colors explained in inset.

We found that 8.3% of substitutions in HARs are recent (Fig. 2a), i.e., not shared with the Sidrón Neandertal or the Neandertal and Denisova genomes. By contrast, 12.4% of substitutions genome-wide are recent. Hence, the fraction of recent alleles in HARs is about 30% smaller than expected from substitutions genome-wide (P_bootstrap_<0.001). For A/T to G/C (weak to strong, W2S) and G/C to A/T (strong to weak, S2W) substitutions, the fractions of recent substitutions in HARs are 6.4% and 11.4%, respectively (Fig. 2a). Thus, recent W2S substitutions are 50% fewer than expected from substitutions genome-wide (P_bootstrap_<0.001), whereas recent S2W substitutions are only 8% fewer and not statistically different from the genome-wide expectation (P_bootstrap_ = 0.09). When W2S and S2W substitutions in HARs are compared to W2S and S2W substitutions genome-wide, we found that the fractions of recent substitutions are in both cases smaller than for substitutions genome-wide (P_bootstrap_<0.001) (Fig. 2a). When positions recovered in the Sidrón Neandertal and the two ancient hominin draft genomes were analyzed separately the same trends are found. However, in the Neandertal genome the difference in the percentage of recent alleles for W2S and S2W changes is larger (Fig. S2). This is likely an artifact resulting from the treatment of the Neandertal libraries with restriction enzymes in order to increase the amount of endogenous DNA [15] (Fig. S3), resulting in a lower recovery of GC-rich HARs in the Neandertal genome. When the published sets of HARs were analyzed separately, the same trends were also seen (Fig. S4 and Table S1, S2) despite the fact that these were ascertained in different ways in each of the publications. We conclude that W2S HAR substitutions are significantly older than S2W substitutions (Mann-Whitney U test (MWU) P<10^−15^). They are also older than substitutions outside HARs and older than substitutions in various functional classes in protein-coding genes (synonymous, non-synonymous, 5′-UTR and 3′-UTR changes) as determined from the Neandertal and Denisova draft genomes (P_bootstrap_ for all comparisons <0.001) (Fig. 2b).

**Figure 3:**
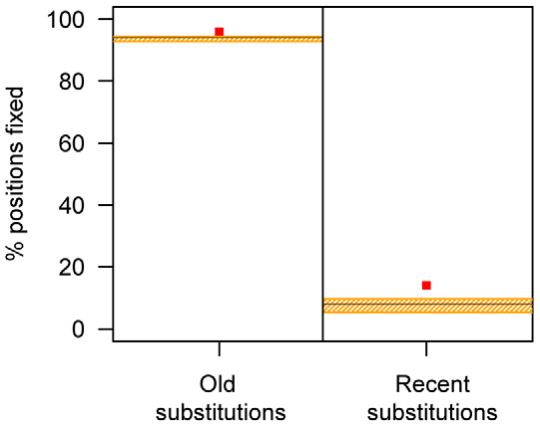
Percentages of fixed positions for old and recent substitutions. The red points show the percentages calculated for HARs, the horizontal black line shows the genome-wide percentage, and the orange shaded areas show 95% confidence intervals for the genome-wide percentage calculated from an empirical distribution after randomly sampling genome-wide HAR-sized elements 1,000 times.

In the HARs, 96% of the old alleles and 16% of the recent alleles are fixed among present-day humans as judged from dbSNP (v. 131). When we sampled 1,000 times ∼2,000 HAR-sized regions from the two archaic genomes 94% of the old alleles are fixed in present-day humans, while only 8% of the recent alleles are fixed. Recent alleles in HARs are thus about twice as likely to be fixed than is expected from genome-wide rates (P_bootstrap_<0.001), indicating that substitutions in HARs tend to fix faster than substitutions elsewhere in the genome (Fig. 3). This applies to both recent W2S and recent S2W alleles in HARs, where 16% and 14%, respectively are fixed and significantly higher than the genome-wide averages (P_bootstrap_<0.001 for both W2S and S2W alleles). The percentages of recent fixations of W2S and S2W alleles were not significantly different from each other (Fisher exact test (FET) p = 0.31).

### Temporal clustering of HAR substitutions

**Figure 4:**
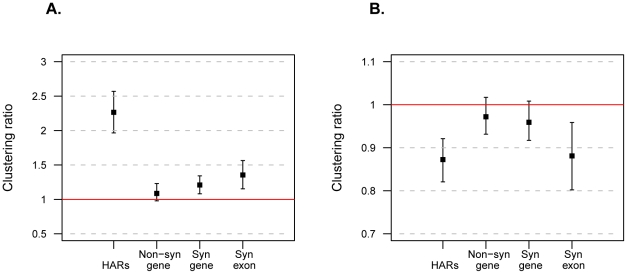
Temporal clustering analysis of HAR substitutions and protein-coding genes. (**A**) Clustering ratios for recent substitutions. HAR ratios computed using all datasets, and changes in genes computed from Neandertal and Denisova genomes. Non-syn gene = non-synonymous substitutions in full-length protein sequences; syn gene = synonymous substitutions in full-length protein sequences; syn exon = synonymous substitutions in individual exon sequences. (**B**) Clustering ratios for old substitutions. In both panels, the red line shows ratio = 1, which indicates an absence of clustering. Error bars are 95% confidence intervals calculated from an empirical distribution after repeating the sample procedure 1,000 times.

To test whether the substitutions within individual HARs occurred clustered in time, we randomly sampled one substitution in each HAR and classified it as old or recent. The HARs were thus divided into two groups. For each group we calculated the percentage of recent alleles using all other positions in the HARs. The percentages of recent alleles in each group were divided by 8.3%, the percentage of recent alleles observed across all HARs. If substitutions in HARs occurred gradually along the human lineage this ratio will not be different from one, whereas if the changes in HARs clustered in time the ratio will be different from one. The ratios were 2.26 and 0.87 for recent and old alleles, respectively (Fig. 4), indicating that if a HAR carries a recent allele, it is about 2.3 times more likely than expected to carry a second recent allele (P_bootstrap_<0.001). In contrast, if it carries an old allele, it is about 15% less likely than expected to carry a second substitution which is recent (P_bootstrap_<0.001). We conclude that substitutions in individual HARs tend to be clustered in time. The same patterns were found when each of different published HAR datasets were analyzed separately (Fig. S5). When we apply the same approach to protein-coding parts of genes using the ancient hominin draft genomes [15], [16] substitutions in HARs were found to be significantly more clustered than both synonymous (MWU at P<10^−15^ for both recent and old alleles) and non-synonymous substitutions (MWU at P<10^−15^ for both recent and old alleles) (Fig. 4). To test if the differences in clustering ratios between HARs and genes was caused by the length difference between the regions studied (short HARs and long genes), we also applied the method to protein-coding exons (median length = 171 bp). Again, the clustering ratios of HARs for both old and new substitutions were significantly more clustered than non-synonymous substitutions within exons (MWU at P<10^−15^ and P<10^−17^, respectively) (Fig. 4).

**Figure 5:**
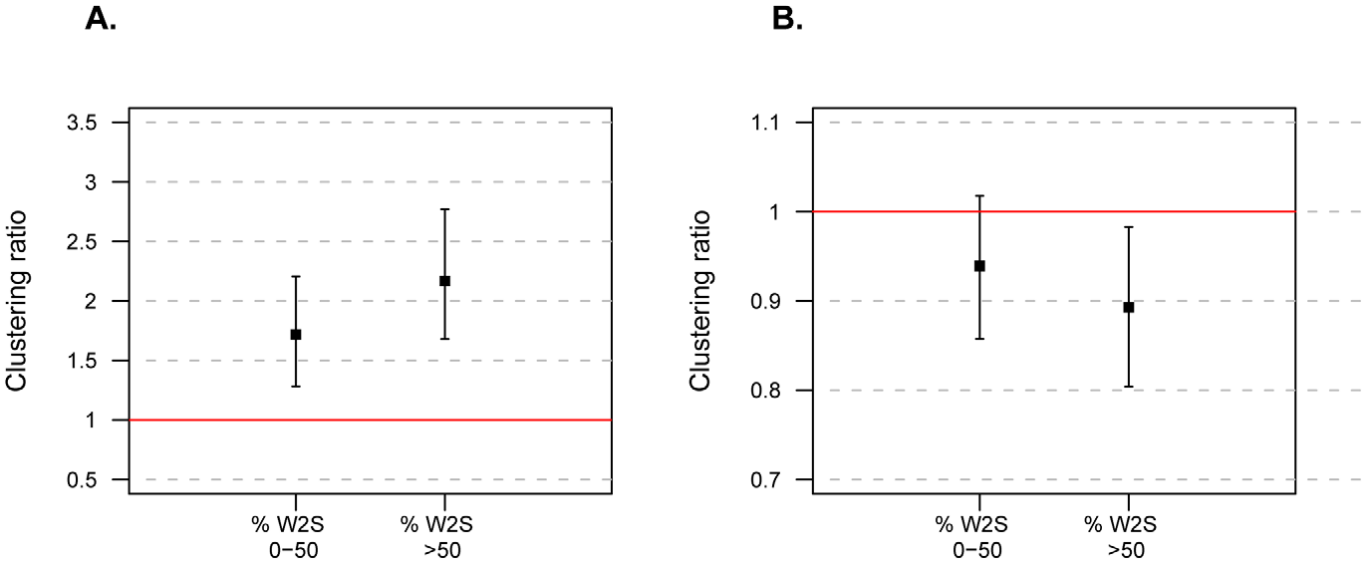
Temporal clustering analysis of HARs. HARs were split in two bins according to their percentage of W2S substitutions: 0 to 50% and >50%. (**A**) Clustering ratios for recent substitutions of each type. (**B**) Clustering ratios for old substitutions of each type. In both panels, the red lines and confidence intervals are calculated as in Figure 4.

To investigate whether both substitutions that are likely to be due to BGC, *i.e.* W2S substitutions, and those that are not, *i.e.* S2W substitutions, cluster in time, we performed the analysis above for HARs where 0–50% of substitutions were of the W2S type (n = 680) and for those where more than 50% were W2S (n = 849). For recent substitutions, the clustering ratios in these two groups were 1.71 and 2.16, respectively. Both ratios were significantly higher than one (P_bootstrap_<0.001), and different from each other (MWU at P<10^−15^) (Fig. 5). For old substitutions, the clustering ratio of the over 50% W2S group was significantly lower than one (P_bootstrap_ = 0.01), whereas the 0–50% group was not (P_bootstrap_ = 0.07). Thus, temporal clustering exists for HARs that are potentially affected by BGC and for those that are not, although clustering is stronger amongst HARs with more W2S substitutions. These findings suggest that substitutions in all HARs, and especially those with some evidence of BGC, fixed surprisingly quickly in the human lineage.

## Discussion

Since Neandertals and Denisovans are sister groups [16] their genomes can be used in concert to gauge when substitutions occurred along the human evolutionary lineage. Here, we complement the analysis of these two published low-coverage genomes with targeted retrieval of 2,613 HARs from a late Neandertal from northwestern Spain [22], [23]. Array capture of HAR regions from this bone enabled us to greatly increase the coverage of human substitutions in HARs by ancient hominin DNA and thereby to validate inferences about the timing of substitutions through comparing multiple data sets. Together these data allow us to estimate which substitutions in HARs are shared with the archaic humans, and to identify those substitutions that are truly specific to the modern human lineage. The “old” substitutions very likely occurred before the population divergence of the ancestors of modern humans from the ancestor of Neandertals and Denisovans, which is estimated to be 270,000 to 440,000 years ago [15]. Those substitutions that we do not observe in the currently available archaic genomes are “recent”, i.e. they existed as polymorphisms in the common ancestor of the archaic hominins and modern humans or occurred in modern humans at a later time. Thus, even a single observation of the ancestral allele allows us to say that an allele is recent. This information can be used to identify potentially interesting HARs that have changed recently in human evolution.

By analyzing evolutionary and sequence characteristics of substitutions in HARs and comparing them to genome-wide substitution patterns, we uncover several interesting aspects of HAR evolution. First, substitutions in HARs tend to be older than substitutions genome-wide or in other categories of functional elements. Second, the rate at which changes in HARs come to fixation is faster than the genome-wide average. In particular recent substitutions in HARs are twice as likely to be fixed compared to substitutions genome-wide. Third, recent substitutions clustered temporally which indicates that HARs that have changed since the common ancestor with Neanderthals and Denisovans (or at least the ones that statistical tests for acceleration can powerfully detect) are particularly fast evolving.

The increased fixation rate could be caused by positive selection or by BGC [9]. To explore the potential impacts of BGC on HAR evolution, we compared evolutionary trends for W2S versus S2W substitutions in HARs. Substitutions of both types are equally likely to be fixed in modern humans and have fairly similar patterns of temporal clustering. However, W2S substitutions in HARs are much less likely to be recent compared to S2W substitutions in HARs, which do not differ significantly from the genome-wide average of 12% recent. This observation suggests that a substantial part of the increased fixation rate of HARS on the human lineage is caused by BGC.

Irrespective of the mechanism that caused the acceleration of substitutions in HARs on the human evolutionary lineage after its divergence from the chimpanzee lineage, at least three, non-mutually exclusive, explanations can contribute to the observations above: (i) The statistical tests used to define HARs have greater power in regions (HARs) that are older since substitutions have then had more time to accumulate; (ii) The effect of negative as well as positive selection and/BGC results in smaller effective population size and thus more rapid drift in HARs, which in turn results in that a larger fraction of substitutions will be fixed and thus shared with archaic humans; (iii) The accelerated evolution in HARs was greater before the divergence of modern humans from the archaic hominins studied to date. Currently, the relative contributions of these three factors to the observations described cannot be reliably estimated since the relative magnitude of the ascertainment bias, drift, BGC and selection are largely unknown.

The sequence conservation of HARs among primates and/or mammals suggests that many of them may be involved in functions that have changed in humans. The classification of substitutions in HARs into old and recent will allow future functional studies to focus on HARs that changed at different times during human evolution. For example, HARs that experienced several changes clustered in time may be enriched for those that lost or altered a function that was previously responsible for their conservation. HARs where changes occurred recently and then rapidly rose to high frequency or fixation in humans may be candidates for having been affected by positive selection since modern humans diverged from archaic humans. We identified 98 such recent substitutions in 69 HARs where present-day humans are fixed for derived variants according to dbSNP (v. 131) (Table S3). One of these HARs overlaps a putative transcriptional enhancer [7] in an intron of the gene *TOX3* (ENSG00000103460), encoding a protein that regulates calcium-dependent transcription in neurons [28]. Such HARs may warrant deeper functional investigation in the future.

## Materials and Methods

### Neandertal DNA extraction and library preparation

We extracted DNA from a Neandertal bone (Sidrón 1253) in our clean room facility as previously described [29]. The bone was excavated in El Sidrón, Spain [23]. During excavation precautionary measures were taken to avoid contaminating the bone with present-day human DNA [25]. In ancient DNA cytosines deaminate to uracils, which can then lead to misidentification of some cytosines for thymines in retrieved sequences [26], [30]. To remove uracils from ancient DNA we treated the extracts with uracil-DNA glycosylase and endonuclease VIII [31]. The extracts then were turned into 454 sequencing libraries as described in [20]. The libraries carry a Neandertal library-specific “key” sequence (TGAC) that only Neandertal libraries produced in our clean room carry [26]. We used 1.4 grams of the Neandertal bone and 475 µl of the extracts for the production of 10 libraries.

### Array design

To capture Neandertal libraries we used Agilent custom 244 K capture arrays. We designed overlapping microarray probes of 60 bases targeting 2613 HARs that were identified in 4 different studies [1], [2], [3], [4]. Probes were tiled every 9 bases across the target regions. Probes containing repetitive elements were discarded [32]. We used the human reference sequence NCBI Build 36.1 (hg18) to design the probes. In addition to probes targeting the HARs, we include probes that target human mitochondrial DNA (mtDNA) sequence in order to estimate the level of present-day human contamination.

### Capture and sequencing of Neandertal libraries

Before capture, the 10 libraries were amplified with PCR as previously described [20] and pooled into one library for a total of 16.8 µg required for the capture. Two serial captures were performed on 244 k Agilent arrays as described in [20]. To sequence the captured library on the Illumina platform we converted the 454 DNA libraries to Solexa sequencing libraries as described in [20]. The converted libraries were sequenced on two lanes of the Illumina GA_II_ sequencer together with PhiX 174 variant spiked in. Manufacturer's sequencing protocol for a paired-end run with 2×76 cycles and v4 chemistry was used except that special sequencing primers were used [20]; the primers were designed so that in the first read the Neandertal-libraries specific “key” sequence was read [20]. DNA sequences are deposited in the European Bioinformatics Institute Sequence Read Archive, with study accession number ERP000837.

### Processing and mapping of Neandertal reads

The sequencing run was processed and base calling was performed as described in [20]. Neandertal reads were aligned to the human reference sequence NCBI Build 36.1 (hg18) using BWA [24]. Reads that aligned to hg18 mtDNA were used to assemble Sidrón mtDNA. The mtDNA was assembled using an iterative mapping assembly program as described in [20]. The identification of the changes that happened in the human lineage was done using whole genome alignments as described in [15], [16]. Using Neandertal overlapping reads, a consensus Neandertal sequence was generated using “mini-contigs” as described in [15], [16].

### Authenticity estimates

El Sidrón 1253 mtDNA differs from almost all (99%) modern human's mtDNA at 130 positions [18]. We estimated the level of modern human mtDNA contamination after microarray capture by classifying each Sidrón mtDNA fragment carrying an informative site as human-like (polluting) or Neandertal-like (clean) [27]. The autosomal contamination estimate was calculated using a likelihood framework [20].

### Statistical analysis

Statistical tests were performed in the R environment (http://www.r-project.org). P_bootstraps_ were calculated using 1000 non-parametric bootstrap iterations.

## Supporting Information

Figure S1 **Autosomal authenticity estimate.** (**A**) Likelihood surface for contamination and heterozygosity as variables. Likelihood ratio computed vs. the maximum likelihood, with colors corresponding to rejection cutoffs using the χ^2^ distribution. (**B**) Constrained likelihood surface, where heterozygosity is held constant – the horizontal axis represents this constant. Confidence intervals plotted using the χ^2^ distribution with one degree of freedom.
(TIF)

Figure S2 **Percentage of recent substitutions in the human lineage for different datasets.** The red line shows the genome-wide average percentage of recent substitutions (12%). Recent substitutions are defined as those found in modern humans but not in the ancient hominins. “Total” refers to the total number of positions recovered in each archaic hominin. “Private” refers to positions only recovered in a given archaic hominin. Error rates for HARs are 95% confidence intervals calculated from an empirical distribution after 1,000 bootstraps. Color code explained in inset.
(TIF)

Figure S3 **Relation between the presence of restriction enzyme motifs used in Neandertal genome enrichment (horizontal axis) and the fraction of human lineage substitutions recovered in HARs (vertical axis).** The horizontal axis shows the minimum number of restriction enzyme motifs present in a HAR. The black points show the median percentage of human lineage substitutions recovered for all HARs. The red discontinuous line shows the median percentage of human lineage substitutions recovered for all HARs independent of the presence of restriction enzyme motifs. (**A**) Sidron. (**B**) Neandertal. (**C**) Denisova.
(TIF)

Figure S4 **Percentage of recent substitutions for different groups of HARs.** The red line shows the genome-wide average percentage of recent substitutions (12%). Recent substitutions are defined as those found in modern humans but not found in the ancient hominins. Error rates for HARs are 95% confidence intervals calculated from an empirical distribution after 1,000 bootstraps. Color code explained in inset. The lower overall percentage of recent substitutions from the Prabhakar dataset is due to the fact that one of the criteria used to identify these HARs required that substitutions be fixed in humans.
(TIF)

Figure S5 **Temporal clustering analysis of HARs from all datasets.** The clustering ratios were calculated independently for each group of HARs. (**A**) Clustering ratios for recent substitutions. (**B**) Clustering ratios for old substitutions. In both panels, the red line shows ratio = 1, which indicates an absence of clustering. Error bars are 95% confidence intervals calculated from an empirical distribution after repeating the sample procedure 1,000 times.
(TIF)

Table S1 **Comparisons between HARs and genome-wide estimate (12%) of new alleles for different HARs' datasets.**
(DOC)

Table S2 **Comparisons between W2S and S2W percentage of new changes for different HARs' datasets.**
(DOC)

Table S3 **HARs with recent human lineage changes fixed in modern humans according to dbSNP 131.** Total refers to the total number of human lineage changes in each HAR. The total number is split in W to S, S to W, and other type of substitutions. The Ensembl gene ID appears alone if the HAR overlap with the gene at least partially. The number after the ‘@’ character shows the distance in base pairs to the nearest gene, when the HAR does not overlap any gene.
(DOC)
