# Supplementary figures and images for: Analysis of Human Accelerated DNA Regions Using Archaic Hominin Genomes
Source: PLoS One. 2012 Mar 7;7(3):e32877. doi: 10.1371/journal.pone.0032877 (PMC3296746; doi:10.1371/journal.pone.0032877)

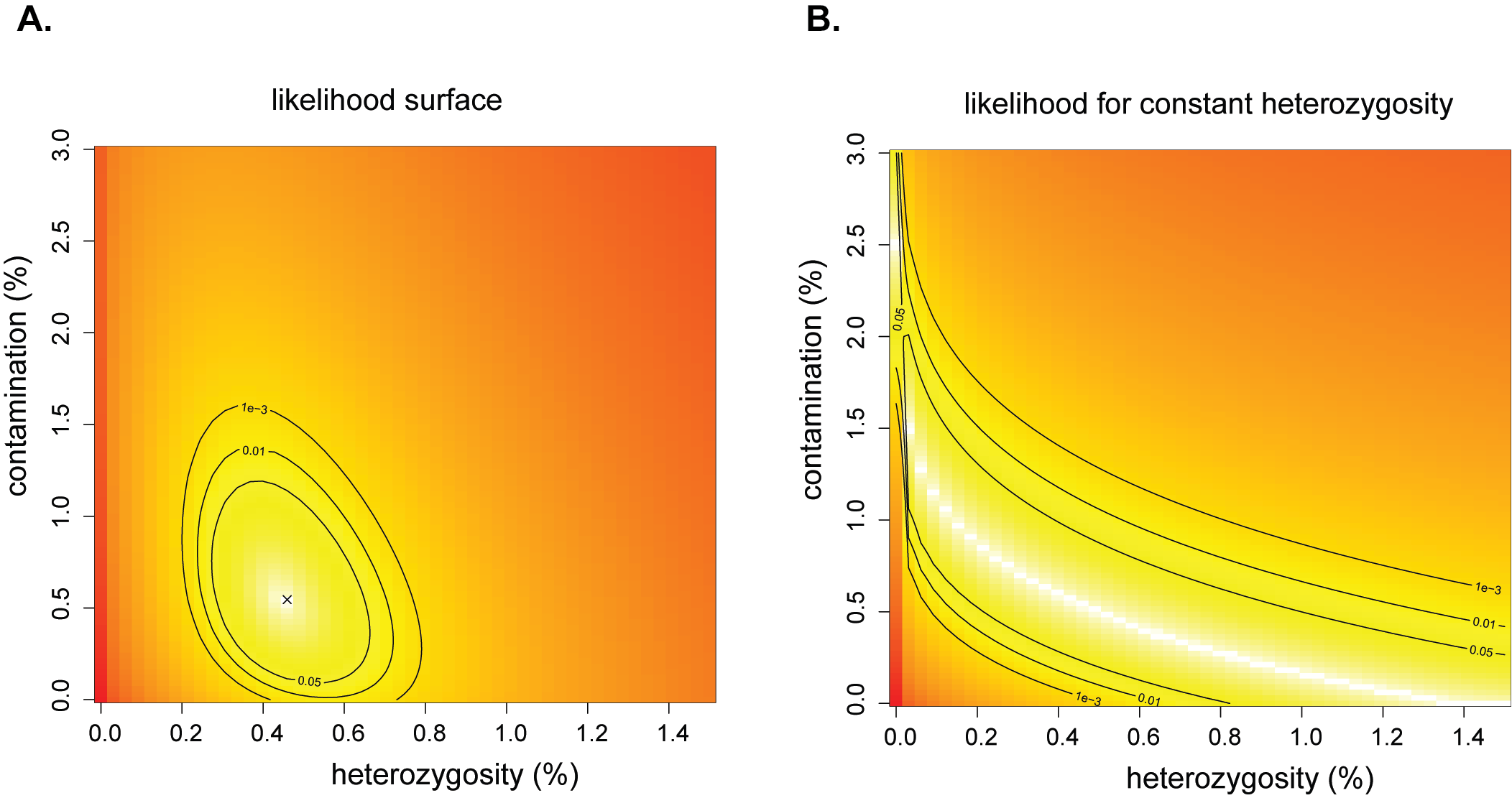

Supplement: Figure S1 — Autosomal authenticity estimate. (A) Likelihood surface for contamination and heterozygosity as variables. Likelihood ratio computed vs. the maximum likelihood, with colors corresponding to rejection cutoffs using the χ2 distribution. (B) Constrained likelihood surface, where heterozygosity is held constant – the horizontal axis represents this constant. Confidence intervals plotted using the χ2 distribution with one degree of freedom. (TIF) [file pone.0032877.s001.tif]

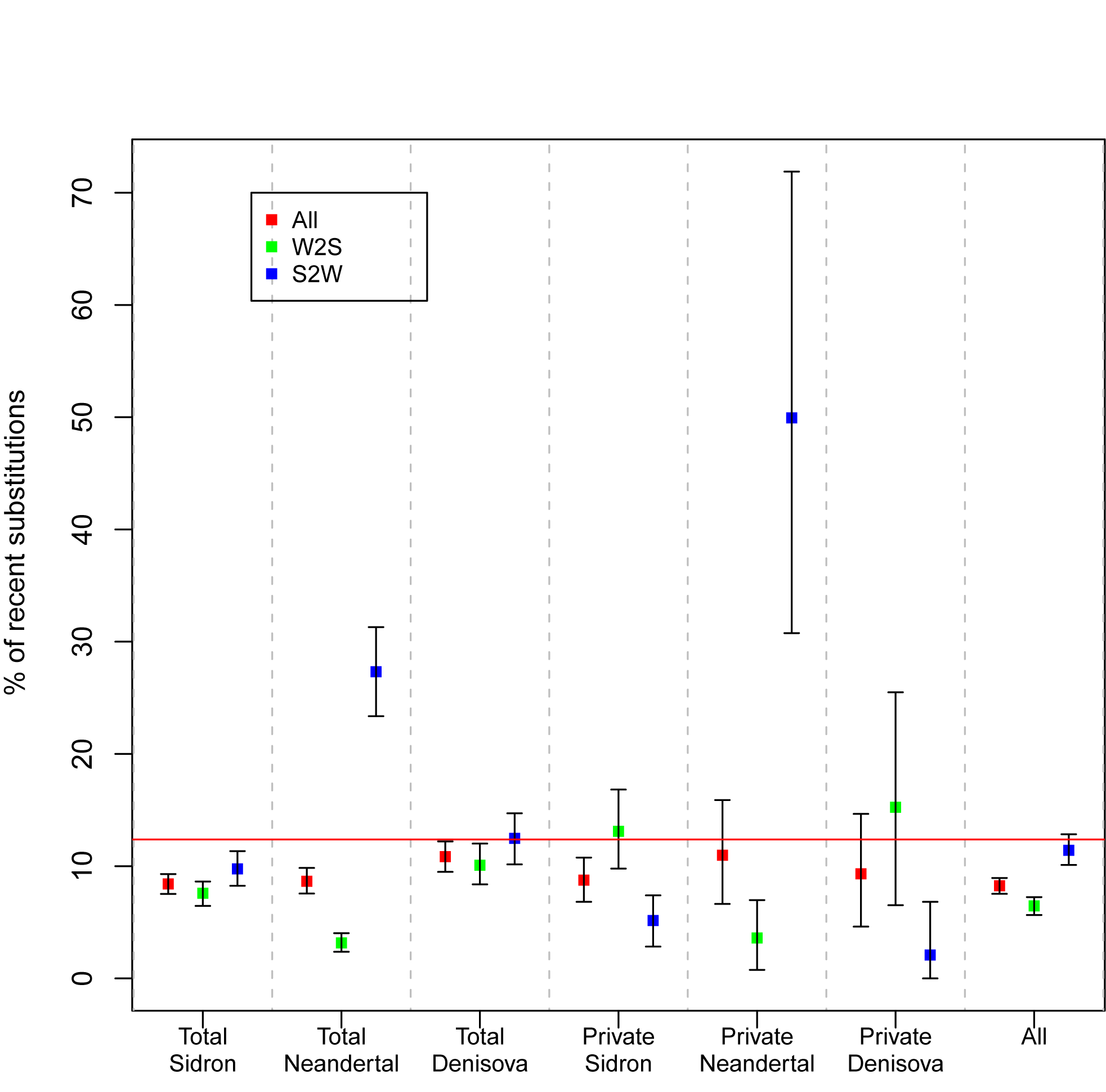

Supplement: Figure S2 — Percentage of recent substitutions in the human lineage for different datasets. The red line shows the genome-wide average percentage of recent substitutions (12%). Recent substitutions are defined as those found in modern humans but not in the ancient hominins. “Total” refers to the total number of positions recovered in each archaic hominin. “Private” refers to positions only recovered in a given archaic hominin. Error rates for HARs are 95% confidence intervals calculated from an empirical distribution after 1,000 bootstraps. Color code explained in inset. (TIF) [file pone.0032877.s002.tif]

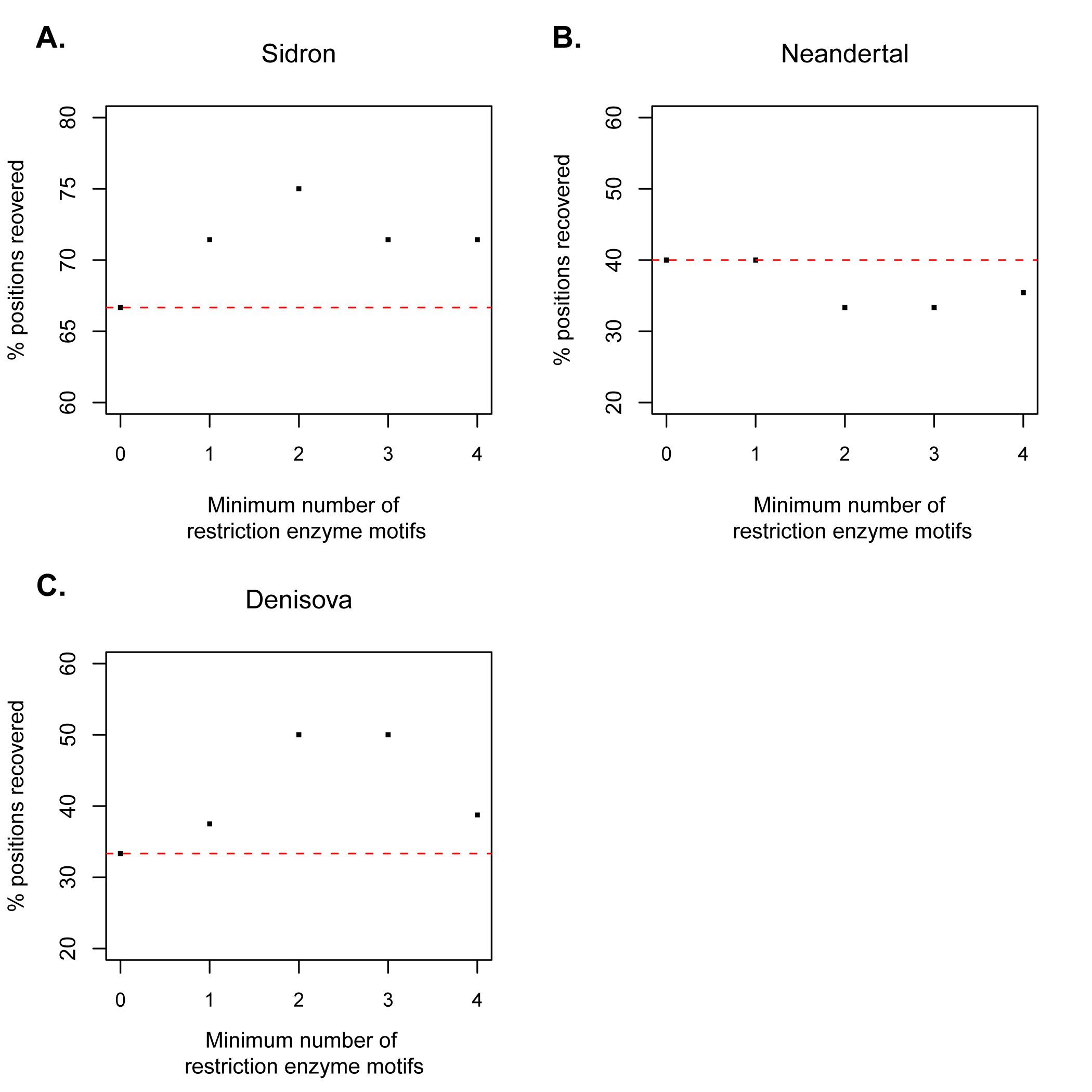

Supplement: Figure S3 — Relation between the presence of restriction enzyme motifs used in Neandertal genome enrichment (horizontal axis) and the fraction of human lineage substitutions recovered in HARs (vertical axis). The horizontal axis shows the minimum number of restriction enzyme motifs present in a HAR. The black points show the median percentage of human lineage substitutions recovered for all HARs. The red discontinuous line shows the median percentage of human lineage substitutions recovered for all HARs independent of the presence of restriction enzyme motifs. (A) Sidron. (B) Neandertal. (C) Denisova. (TIF) [file pone.0032877.s003.tif]

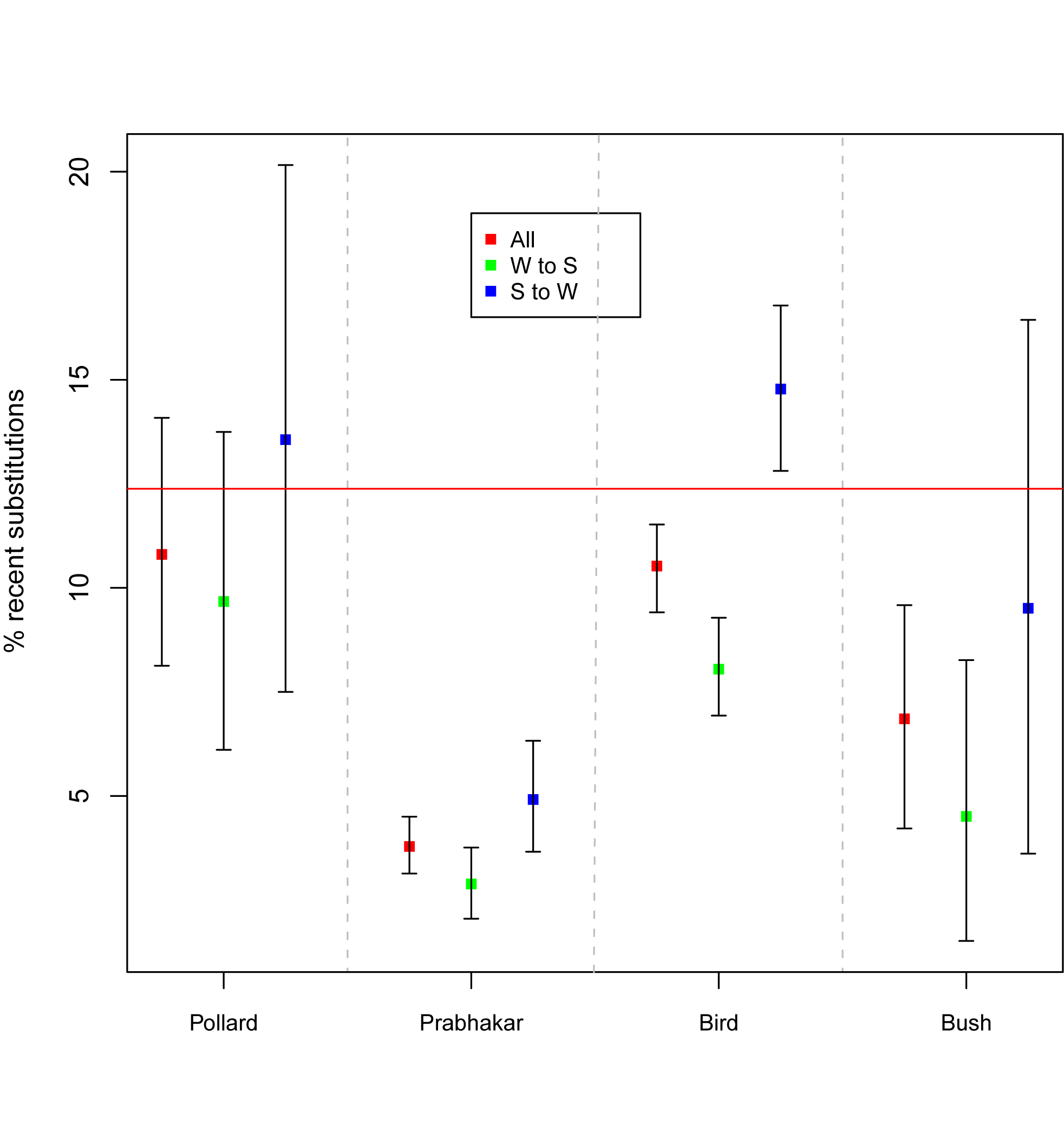

Supplement: Figure S4 — Percentage of recent substitutions for different groups of HARs. The red line shows the genome-wide average percentage of recent substitutions (12%). Recent substitutions are defined as those found in modern humans but not found in the ancient hominins. Error rates for HARs are 95% confidence intervals calculated from an empirical distribution after 1,000 bootstraps. Color code explained in inset. The lower overall percentage of recent substitutions from the Prabhakar dataset is due to the fact that one of the criteria used to identify these HARs required that substitutions be fixed in humans. (TIF) [file pone.0032877.s004.tif]

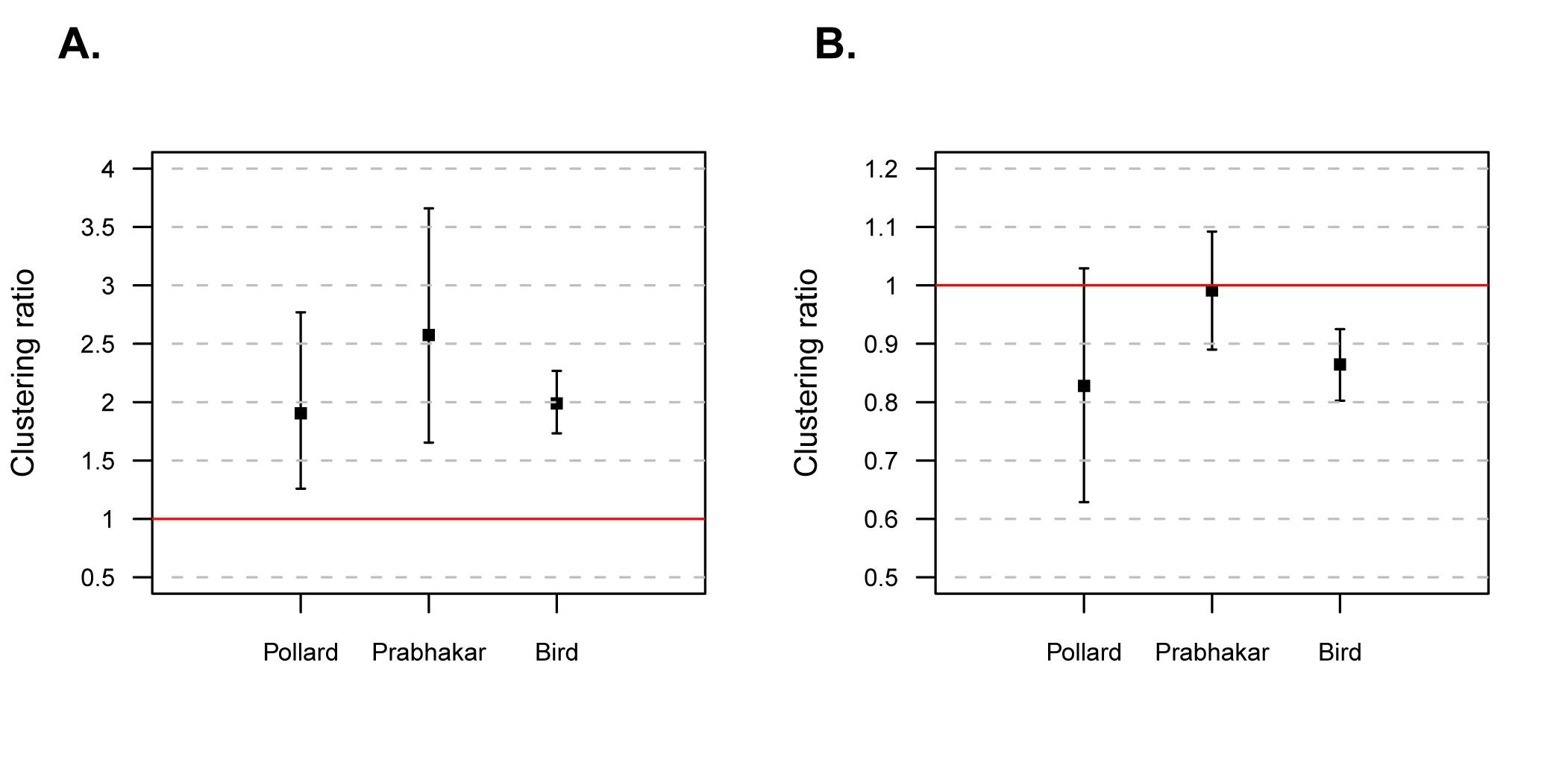

Supplement: Figure S5 — Temporal clustering analysis of HARs from all datasets. The clustering ratios were calculated independently for each group of HARs. (A) Clustering ratios for recent substitutions. (B) Clustering ratios for old substitutions. In both panels, the red line shows ratio = 1, which indicates an absence of clustering. Error bars are 95% confidence intervals calculated from an empirical distribution after repeating the sample procedure 1,000 times. (TIF) [file pone.0032877.s005.tif]
