# Supplementary material for: Analysis of Human Accelerated DNA Regions Using Archaic Hominin Genomes
Source: PLoS One. 2012 Mar 7;7(3):e32877. doi: 10.1371/journal.pone.0032877 (PMC3296746; doi:10.1371/journal.pone.0032877)
Supplement: Table S1 — Comparisons between HARs and genome-wide estimate (12%) of new alleles for different HARs' datasets. (DOC) [file pone.0032877.s006.doc]

**Table S1.** Comparisons between HARs and genome-wide estimate (12%) of new alleles for different HARs´ datasets.

| **Dataset** | **Type of changes** | **P_bootstrap_** |
| --- | --- | --- |
| Pollard et al. | All | 0.157 |
| Prabhakar et al. | All | < 0.001 |
| Bird et al. | All | 0.001 |
| Bush et al. | All | < 0.001 |
| Pollard et al. | W2S | 0.095 |
| Prabhakar et al. | W2S | < 0.001 |
| Bird et al. | W2S | < 0.001 |
| Bush et al. | W2S | < 0.001 |
| Pollard et al. | S2W | 0.632 |
| Prabhakar et al. | S2W | < 0.001 |
| Bird et al. | S2W | 0.009 |
| Bush et al. | S2W | 0.185 |
