# Supplementary material for: Analysis of Human Accelerated DNA Regions Using Archaic Hominin Genomes
Source: PLoS One. 2012 Mar 7;7(3):e32877. doi: 10.1371/journal.pone.0032877 (PMC3296746; doi:10.1371/journal.pone.0032877)
Supplement: Table S2 — Comparisons between W2S and S2W percentage of new changes for different HARs' datasets. (DOC) [file pone.0032877.s007.doc]

**Table S2.** Comparisons between W2S and S2W percentage of new changes for different HARs´ datasets.

| **Dataset** | **P-value** |
| --- | --- |
| Pollard et al. | < 10^-15^ |
| Prabhakar et al. | < 10^-15^ |
| Bird et al. | < 10^-15^ |
| Bush et al. | < 10^-15^ |
