# Supplementary material for: Analysis of Human Accelerated DNA Regions Using Archaic Hominin Genomes
Source: PLoS One. 2012 Mar 7;7(3):e32877. doi: 10.1371/journal.pone.0032877 (PMC3296746; doi:10.1371/journal.pone.0032877)
Supplement: Table S3 — HARs with recent human lineage changes fixed in modern humans according to dbSNP 131. Total refers to the total number of human lineage changes in each HAR. The total number is split in W to S, S to W, and other type of substitutions. The Ensembl gene ID appears alone if the HAR overlap with the gene at least partially. The number after the ‘@’ character shows the distance in base pairs to the nearest gene, when the HAR does not overlap any gene. (DOC) [file pone.0032877.s008.doc]

**Table S3.** HARs with recent human lineage changes fixed in modern humans according to dbSNP 131. Total refers to the total number of human lineage changes in each HAR. The total number is split in W to S, S to W, and other type of substitutions. The Ensembl gene ID appears alone if the HAR overlap with the gene at least partially. The number after the ‘@’ character shows the distance in base pairs to the nearest gene, when the HAR does not overlap any gene.

| **ID** | **Total** | **W to S** | **S to W** | **Other** | **Closest or overlapping Ensembl Gene** |
| --- | --- | --- | --- | --- | --- |
| Prabhakar_849; | 5 | 3 | 2 | 0 | ENSG00000108018 |
| Bird_435; | 3 | 0 | 1 | 2 | ENSG00000162825@-119460 |
| Bird_34; | 3 | 0 | 1 | 2 | ENSG00000162825@-299708 |
| Bird_29; | 3 | 1 | 2 | 0 | ENSG00000197261@-901956 |
| Bird_134; | 3 | 0 | 3 | 0 | ENSG00000218796:ENSG00000065809 |
| Bird_1129; | 3 | 1 | 1 | 1 | ENSG00000205565@-102522 |
| Bird_1128; | 3 | 1 | 1 | 1 | ENSG00000214997@-103300 |
| Prabhakar_859; | 2 | 0 | 1 | 1 | ENSG00000215421 |
| Prabhakar_670; | 2 | 1 | 1 | 0 | ENSG00000148655 |
| Prabhakar_662; | 2 | 2 | 0 | 0 | ENSG00000203566@-50842 |
| Prabhakar_52;Bird_87; | 2 | 2 | 0 | 0 | ENSG00000182718 |
| Bird_924; | 2 | 2 | 0 | 0 | ENSG00000196775@-334085 |
| Bird_624; | 2 | 1 | 1 | 0 | ENSG00000219045@-1235834 |
| Bird_465; | 2 | 1 | 0 | 1 | ENSG00000143702 |
| Bird_437; | 2 | 0 | 1 | 1 | ENSG00000212544@-62215 |
| Bird_24; | 2 | 0 | 1 | 1 | ENSG00000214392@-270811 |
| Bird_21; | 2 | 0 | 1 | 1 | ENSG00000215270@-34563 |
| Bird_18; | 2 | 1 | 1 | 0 | ENSG00000219561 |
| Bird_1329; | 2 | 0 | 0 | 2 | ENSG00000220358@-370481 |
| Bird_1015; | 2 | 2 | 0 | 0 | ENSG00000175161 |
| Prabhakar_977; | 1 | 0 | 1 | 0 | ENSG00000221289@-733194 |
| Prabhakar_962; | 1 | 0 | 1 | 0 | ENSG00000149654 |
| Prabhakar_865; | 1 | 1 | 0 | 0 | ENSG00000173068 |
| Prabhakar_835; | 1 | 0 | 0 | 1 | ENSG00000215248@-133636 |
| Prabhakar_834; | 1 | 0 | 1 | 0 | ENSG00000217171@-670100 |
| Prabhakar_800; | 1 | 0 | 1 | 0 | ENSG00000200028@-829196 |
| Prabhakar_686; | 1 | 0 | 0 | 1 | ENSG00000162971@-217828 |
| Prabhakar_632;Bird_642; | 1 | 1 | 0 | 0 | ENSG00000210310@-146522 |
| Prabhakar_609; | 1 | 0 | 1 | 0 | ENSG00000099139 |
| Prabhakar_561; | 1 | 0 | 0 | 1 | ENSG00000205133@-321342 |
| Prabhakar_532; | 1 | 1 | 0 | 0 | ENSG00000107249 |
| Prabhakar_528; | 1 | 0 | 0 | 1 | ENSG00000100836@-45508 |
| Prabhakar_4;Bush28; | 1 | 1 | 0 | 0 | ENSG00000221321@-39989 |
| Prabhakar_410; | 1 | 1 | 0 | 0 | ENSG00000112699 |
| Prabhakar_333; | 1 | 0 | 0 | 1 | ENSG00000124249@-80331 |
| Prabhakar_261;Bush61; | 1 | 0 | 0 | 1 | ENSG00000148484 |
| Prabhakar_213; | 1 | 0 | 0 | 1 | ENSG00000214283@-401313 |
| Prabhakar_192; | 1 | 0 | 1 | 0 | ENSG00000223148@-1275154 |
| Prabhakar_136; | 1 | 0 | 1 | 0 | ENSG00000103460 |
| Pollard_88;Prabhakar_16;Bird_13; | 1 | 1 | 0 | 0 | ENSG00000164253@-102672 |
| Pollard_82; | 1 | 1 | 0 | 0 | ENSG00000207360@-240499 |
| Pollard_61; | 1 | 1 | 0 | 0 | ENSG00000008196@-176835 |
| Pollard_56;Bird_173; | 1 | 1 | 0 | 0 | ENSG00000132334@-960005 |
| Pollard_200; | 1 | 0 | 1 | 0 | ENSG00000210754@-708397 |
| Pollard_108; | 1 | 1 | 0 | 0 | ENSG00000210754@-373209 |
| Bird_919; | 1 | 1 | 0 | 0 | ENSG00000216393 |
| Bird_795; | 1 | 0 | 1 | 0 | ENSG00000202250@-353921 |
| Bird_75; | 1 | 0 | 1 | 0 | ENSG00000219892@-122164 |
| Bird_748; | 1 | 0 | 0 | 1 | ENSG00000222244@-1009917 |
| Bird_721; | 1 | 1 | 0 | 0 | ENSG00000183458@-1537567 |
| Bird_685; | 1 | 1 | 0 | 0 | ENSG00000196102@-158238 |
| Bird_646; | 1 | 1 | 0 | 0 | ENSG00000211866 |
| Bird_527; | 1 | 0 | 1 | 0 | ENSG00000148655 |
| Bird_473; | 1 | 0 | 1 | 0 | ENSG00000187147 |
| Bird_428; | 1 | 1 | 0 | 0 | ENSG00000106348@-122275 |
| Bird_356; | 1 | 1 | 0 | 0 | ENSG00000173068 |
| Bird_339; | 1 | 1 | 0 | 0 | ENSG00000106610@-1016652 |
| Bird_215; | 1 | 0 | 1 | 0 | ENSG00000213547@-134087 |
| Bird_173; | 1 | 0 | 0 | 1 | ENSG00000132334@-960011 |
| Bird_17; | 1 | 1 | 0 | 0 | ENSG00000198277@-140571 |
| Bird_169; | 1 | 0 | 1 | 0 | ENSG00000137970@-300830 |
| Bird_146; | 1 | 1 | 0 | 0 | ENSG00000214384@-90714 |
| Bird_145; | 1 | 1 | 0 | 0 | ENSG00000214392@-214906 |
| Bird_1332; | 1 | 0 | 1 | 0 | ENSG00000047230 |
| Bird_1224; | 1 | 1 | 0 | 0 | ENSG00000168303@-1286097 |
| Bird_1143; | 1 | 0 | 1 | 0 | ENSG00000164418 |
| Bird_1115; | 1 | 0 | 0 | 1 | ENSG00000221721@-3108896 |
| Bird_1081; | 1 | 1 | 0 | 0 | ENSG00000209177@-1210477 |
| Bird_105; | 1 | 0 | 1 | 0 | ENSG00000169379 |
